# Leflunomide as a therapeutic alternative to methotrexate as a glucocorticoid-sparing agent in polymyalgia rheumatica

**DOI:** 10.1093/rap/rkae047

**Published:** 2024-03-28

**Authors:** Miguel A González-Gay, Elena Heras-Recuero, Teresa Blázquez-Sánchez, Santos Castañeda, Raquel Largo

**Affiliations:** Division of Rheumatology, IIS-Fundación Jiménez Díaz, Madrid, Spain; Medicine and Psychiatry Department, Universidad of Cantabria, Santander, Spain; Division of Rheumatology, IIS-Fundación Jiménez Díaz, Madrid, Spain; Division of Rheumatology, IIS-Fundación Jiménez Díaz, Madrid, Spain; Division of Rheumatology, Hospital Universitario de La Princesa, IIS-Princesa, Madrid, Spain; Division of Rheumatology, IIS-Fundación Jiménez Díaz, Madrid, Spain

**This editorial refers to ‘Effectiveness of methotrexate and leflunomide as corticoid-sparing drugs in patients with polymyalgia rheumatica’ by Juan Pablo Vinicki *et al.*, 2024; https://doi.org/10.1093/rap/rkae033.**

Glucocorticoids constitute the cornerstone of therapy in the management of polymyalgia rheumatica (PMR) [1]. Nevertheless, their use in a disease that affects people ≥50 years of age is associated with significant morbidity [2]. This is closely related to the occurrence of frequent relapses when glucocorticoids are tapered [3], resulting in a prolonged duration of glucocorticoid treatment. Consequently, the efficacy of glucocorticoid-sparing agents in the management of PMR patients, in particular in those who have comorbidities or who experience relapses, is a matter of great interest. Although a recent study highlighted the role of the anti-IL-6 receptor sarilumab in sustaining remission and reducing the cumulative glucocorticoid dose in PMR patients with a relapse during glucocorticoid tapering [4], most studies on this issue have focused on the utility of conventional synthetic disease-modifying antirheumatic drugs (csDMARDs). Regarding this matter, Vinicki *et al.* [5] performed an observational study to assess the outcomes of PMR patients treated in daily clinical practice with either methotrexate (MTX) or leflunomide (LEF) as a glucocorticoid-sparing agent. They reported superiority of LEF for achieving remission and glucocorticoid discontinuation. The study provides valuable insights, particularly given the limited available data on the use of LEF in PMR and its comparison with MTX.

MTX stands out as the most used csDMARD in the treatment of PMR, functioning as a glucocorticoid-sparing agent. The typical initial dosage of MTX in PMR falls within the range of 10–15 mg/week. Despite contradictory studies, the overall consensus suggests that the combination of MTX with a prednisone regimen offers advantages for individuals at increased risk of glucocorticoid-related adverse effects [6]. A comprehensive review of the literature showed that this combination produces a lower incidence of adverse effects compared with the use of prednisone alone [7]. Given these findings, the 2015 EULAR/ACR recommendations for PMR management included a conditional recommendation to consider the early incorporation of MTX, particularly for patients facing a high risk of relapse and/or prolonged therapy. This recommendation extended to cases involving risk factors, comorbidities and/or concomitant medications where glucocorticoid-related adverse events are more likely [8]. MTX was suggested for use in the follow-up of patients experiencing a relapse with an inadequate response to glucocorticoids or encountering adverse events related to glucocorticoid treatment [8].

The potential hepatotoxicity of LEF in the elderly may raise concerns, given the black box warning for liver injury associated with this drug. This caution is particularly relevant for aging individuals with pre-existing liver disease, elevated liver enzymes or those concurrently using medications known to cause liver injury [6]. Probably due to this fact, there is limited information on LEF in PMR, with only two case series available. In this context, Diamantopoulos *et al.* [9] retrospectively evaluated a small series that included difficult-to-treat patients with giant cell arteritis (GCA) (*n* = 11) and PMR (*n* = 12). Patients started with 10 mg/day LEF and the dose was increased to 20 mg if the clinical response was insufficient or according to the judgment of the treating physician. Six patients (26%; three PMR and three GCA) discontinued treatment due to side effects. However, no serious adverse events requiring hospitalization were recorded. A total of 5 of the 23 patients (2 PMR and 3 GCA) discontinued treatment due to remission after a mean period of 10.2 months [9]. In the PMR group, a 6 mg/dl reduction in CRP levels and a 34.2% reduction in the prednisolone dose were achieved [9]. In another study, Adizie *et al.* [10] assessed the efficacy and adverse effects of LEF in 9 patients with GCA and 14 with PMR. All patients had difficulty in tapering the prednisolone dose and three did not responded to optimal doses of MTX. An initial dose of 10 mg/day of LEF was administered to them, ranging from 3 to 9 months after initiating the glucocorticoid treatment. The LEF dose was increased to 10/20 mg on alternate days (five patients) or 20 mg/day (two patients) if needed, as per clinical and biochemical response. LEF was well tolerated in all patients except three. All patients with GCA and 13 of 14 with PMR showed a complete or partial response to LEF. Glucocorticoids were discontinued in 9 and reduced in 12 of the 23 patients [10]. Despite potential limitations of the study due to its open, non-randomized design, lack of a control group and small number of patients, the results indicated that LEF is well tolerated in GCA and PMR patients, with good clinical response, favouring glucocorticoid tapering.

Vinicki *et al.* [5] evaluated 143 PMR patients treated with MTX (median dose 15 mg/week) and 43 who received LEF (20 mg/day, fixed dose). The glucocorticoid dose at baseline and tapering was at the discretion of the treating physician and were not pre-set. Sampling of the centres was not randomized. The patients underwent a follow-up period of at least 3 months from the initiation of the conventional DMARD. Glucocorticoid withdrawal was achieved more commonly in LEF- (72%) than in MTX-treated patients (39%). However, the study lacked a long-term follow-up, especially in the LEF group. Of major concern could be the fact that there were differences in ESR and CRP levels between groups at the time of recruitment. Although the authors argue that they do not believe that differences between the groups would have influenced the results, the MTX group seemed to have more severe disease as evidenced by higher CRP/ESR at diagnosis and baseline, higher prednisone dose at baseline and longer disease duration. Furthermore, many of the MTX-treated patients received ≤15 mg/week, which means that its potential inferiority compared with LEF could be explained by underdosing. Despite these potential limitations, the study has clinical interest. In this regard, the time until prednisone discontinuation was shorter in the LEF-treated patients (median 4.7 months *vs* 31.8 months in the MTX-treated patients). Moreover, in the multivariate analysis, the probability of remission was significantly higher with LEF therapy.

In conclusion, despite the limitations, this study by Vinicki *et al.* [5] provides valuable information on the use of LEF in PMR. As the authors suggest, further studies including larger series of PMR patients followed prospectively are needed to support these promising data on the efficacy of LEF in PMR.

## Data Availability

All data relevant to this editorial are included in the article. Additional information is available upon request.
